# On the processes generating latitudinal richness gradients: identifying diagnostic patterns and predictions

**DOI:** 10.3389/fgene.2014.00420

**Published:** 2014-12-02

**Authors:** Allen H. Hurlbert, James C. Stegen

**Affiliations:** ^1^Department of Biology, University of North CarolinaChapel Hill, NC, USA; ^2^Curriculum for the Environment and Ecology, University of North CarolinaChapel Hill, NC, USA; ^3^Pacific Northwest National LaboratoryRichland, WA, USA

**Keywords:** biodiversity, disturbance, diversification, latitudinal gradient, simulation, speciation rate, species richness, zero sum

## Abstract

We use a simulation model to examine four of the most common hypotheses for the latitudinal richness gradient and identify patterns that might be diagnostic of those four hypotheses. The hypotheses examined include (1) tropical niche conservatism, or the idea that the tropics are more diverse because a tropical clade origin has allowed more time for diversification in the tropics and has resulted in few species adapted to extra-tropical climates. (2) The ecological limits hypothesis suggests that species richness is limited by the amount of biologically available energy in a region. (3) The speciation rates hypothesis suggests that the latitudinal gradient arises from a gradient in speciation rates. (4) Finally, the tropical stability hypothesis argues that climatic fluctuations and glacial cycles in extratropical regions have led to greater extinction rates and less opportunity for specialization relative to the tropics. We found that tropical niche conservatism can be distinguished from the other three scenarios by phylogenies which are more balanced than expected, no relationship between mean root distance (MRD) and richness across regions, and a homogeneous rate of speciation across clades and through time. The energy gradient, speciation gradient, and disturbance gradient scenarios all produced phylogenies which were more imbalanced than expected, showed a negative relationship between MRD and richness, and diversity-dependence of speciation rate estimates through time. We found that the relationship between speciation rates and latitude could distinguish among these three scenarios, with no relation expected under the ecological limits hypothesis, a negative relationship expected under the speciation rates hypothesis, and a positive relationship expected under the tropical stability hypothesis. We emphasize the importance of considering multiple hypotheses and focusing on diagnostic predictions instead of predictions that are consistent with multiple hypotheses.

“The reason every one of you is telling it differently is because each one of you touched a different part of the elephant. So, actually the elephant has all the features you mentioned.”

—“Elephant and the blind men,” Jain Stories, JainWorld.com

## INTRODUCTION

While biologists have generated a variety of hypotheses to explain the teeming diversity of life in the tropics relative to more temperate regions, there has been less progress in ruling out or agreeing on the primary processes responsible for biodiversity patterns. Because such patterns result from a mixture of ecological and evolutionary processes playing out over space and time, and because these patterns may be assessed at multiple spatial and temporal scales and for radically different taxonomic groups, it is unsurprising that different investigators have emphasized the importance of different processes. In some respects, biodiversity patterns and their study resemble the allegorical elephant that is examined by multiple blind men, each coming to radically different conclusions about the nature of their study subject. Independent investigations into different aspects of biodiversity patterns have resulted in conflicting conclusions about underlying processes.

There are two primary ways in which biodiversity research resembles efforts of the apocryphal blind men. First, the narrow focus of most studies on a single process, pattern, region, or taxonomic group undermines our ability to properly evaluate and distinguish among hypotheses and precludes understanding of how processes change across systems—the blind man who attempts to characterize an elephant based entirely on feeling the tail is doomed to fail. Although there have been several promising efforts toward broader integration (e.g., Hillebrand, 2004; McGill, 2010; Jansson et al., 2013), ecologists and evolutionary biologists would more rapidly advance biodiversity science by simultaneously evaluating multiple hypotheses that make predictions about multiple patterns associated with biodiversity gradients. After all, if the elephant represents the truth about how biodiversity gradients originate and are maintained, then an appreciation for that truth can only be obtained by recognizing that it must simultaneously explain the tail, ear, legs, body, trunk, and tusks.

Second, many studies make inferences based on observations that might be consistent with a particular hypothesis, but that are neither unique to nor sufficient for that hypothesis. One blind man based his conclusion that the elephant was a rope on the fact that he felt something long, narrow, and frayed at the end. Had he started with a set of *a priori* hypotheses and their associated predictions, it would have been clear that multiple hypotheses are consistent with such a narrow set of observations. Informed by *a priori* predictions, the blind man would have characterized a different set of features that would collectively distinguish among his hypotheses. In a biodiversity context, the observation that species richness is positively correlated with net primary productivity is consistent with an argument based on energetic or ecological limits (e.g., Wright, 1983), but is also consistent with explanations based on other causal variables that might correlate with productivity (e.g., rates of speciation, time in a region, degree of similarity to ancestral environments). To distinguish among a set of hypotheses one must test predictions that are diagnostic; testing many necessary but non-diagnostic predictions is necessary but not sufficient.

Our broad goal here is to continue developing an approach that facilitates the integration of simulation models with empirical data to enable the advancement of biodiversity science into the paradigm of multi-hypothesis/multi-prediction evaluation (e.g., Stegen and Hurlbert, 2011; Stegen et al., 2012; Hurlbert and Stegen, 2014). We specifically develop multi-pattern predictions from four hypotheses that have been proposed to explain patterns of species richness. The tropical niche conservatism hypothesis suggests that tropical regions have more species because descendants of a tropical ancestor will all tend to have tropical environmental tolerances, and diversity outside the tropics is hence constrained by both successful colonization and limited time for diversification (Wiens et al., 2010; Romdal et al., 2013). The ecological limits hypothesis (“energy gradient”) suggests that tropical regions are most diverse because of a greater energetic capacity to support viable species populations (Wright, 1983; Hurlbert and Stegen, 2014). The evolutionary rates hypothesis (“speciation gradient”) suggests that evolutionary rates are faster in the tropics whether because of the kinetic effects of temperature (Rohde, 1992; Allen and Gillooly, 2006), the increased importance of biotic interactions (Schemske, 2009), or increased area (Rosenzweig, 1995). Finally, the tropical stability hypothesis (“disturbance gradient”) suggests that tropical environments have been less susceptible to major disturbances such as repeated glaciations that have led to higher extinction rates in temperate to polar latitudes (Brown and Lomolino, 1998; Weir and Schluter, 2007).

To generate multi-pattern predictions associated with the above hypotheses, we build on a previous spatial simulation model of diversification and dispersal across a broad environmental gradient (Hurlbert and Stegen, 2014). By incorporating into the model different assumptions that correspond to the above hypotheses we evaluate the ability of multiple predicted patterns to diagnose underlying process.

While the simulated scenarios differ in critical ways, they all share several key components that we view as likely features of any realistic diversification process across a broad gradient. These include (1) niche conservatism, where descendants have traits that are similar to their ancestors with respect to environmental tolerances (Wiens and Graham, 2005); (2) environmental filtering (or “selection,” Vellend, 2010), where species with traits poorly suited to their environment will have reduced performance and lower population sizes compared to species with traits well-suited to their environment; and (3) stochastic extinction that occurs with a probability that declines exponentially with increasing population size (Lande et al., 2003). While we examine one scenario in which there are no energetic limits and individuals and species may accumulate exponentially and indefinitely through time (the “pure niche conservatism” scenario), the rest of the simulations impose a zero sum energetic constraint such that increases in abundance or biomass by one species must be offset by a collective decrease across all other species (Hurlbert and Stegen, 2014).

As a starting point for diagnosing the diversification scenario underlying a given dataset, we examined secondary biodiversity patterns and metrics highlighted in Hurlbert and Stegen (2014). However, the consideration of additional diversification scenarios here requires additional patterns to differentiate them. As such, we used a recently developed analysis framework (Rabosky, 2014) for characterizing diversification rates through time and across the simulated phylogenies emerging from our four scenarios. We present model outputs as a collection of patterns that appear to differentiate among diversification scenarios associated with the tropical niche conservatism, ecological limits, evolutionary rates, and tropical stability hypotheses.

## MATERIALS AND METHODS

We utilize a simulation model of diversification and dispersal along a one-dimensional spatial gradient spanning 10 adjacent regions from the warm tropics to the cooler temperate zone as described in Hurlbert and Stegen (2014). Briefly, a simulation begins with a single species originating within a region at either the tropical or temperate end of the gradient, which achieves a regional population size that is determined in part by the match between the regional environment and the species’ intrinsic environmental optimum. Each species has a fixed per-individual probability of spawning a daughter species which inherits the environmental optimum of its parent with some small amount of variation, reflecting strong niche conservatism. Each species also has a fixed per-individual probability of dispersing to adjacent regions. Members of the diversifying clade are envisioned to use a common pool of limiting resources such that a zero sum energy constraint is a reasonable assumption (Hurlbert and Stegen, 2014). Each region can therefore support some maximum number of individuals summed across species. As the number of species increases in a region, the average population size decreases. Finally, extinction occurs stochastically with a per species probability that is a negative exponential function of population size.

We examined three different zero-sum scenarios that have the potential to influence latitudinal gradients in species richness (**Table 1**). In each scenario, one key parameter varied across the spatial gradient, and all three scenarios were able to support the same global number of individuals. Under the “energy gradient” scenario, the total number of individuals that could be supported in a region increased linearly from 4,000 in the temperate zone to 40,000 in the tropics. The 10-fold increase in carrying capacity across the gradient is similar to the roughly 10-fold increase in net primary productivity across the latitudinal gradient at coarse resolutions (Kucharik et al., 2000). Under the “speciation gradient” scenario, the per-capita probability of speciation increased linearly from 3 × 10^-7^ in the temperate zone to 3 × 10^-6^ in the tropics. This 10-fold gradient in speciation rate is at the upper end of estimates for how speciation or net diversification rates might vary across latitudes (Allen et al., 2006; Rolland et al., 2014). Under the “disturbance gradient” scenario, disturbance events occurred with a regular frequency leading to extinctions, but the magnitude of disturbance events increased linearly from 75% of all individuals being killed per event in the tropics to 99% in the temperate zone. We chose a relatively high value of disturbance for tropical regions because it is increasingly recognized that tropical regions have been impacted by global climate fluctuations (Colinvaux et al., 1997). Nevertheless, in our disturbance gradient scenario, such impacts are still less than would be expected by physical displacement of species by ice sheets at high latitudes. All zero-sum simulations were run for 100,000 time steps, or approximately five times longer than it took for equilibrial richness gradients to emerge, and 10 replicate simulations were run for each scenario.

**Table 1:** Parameter values used in the four simulation scenarios presented in this analysis.

Parameter	Pure niche conservatism	Energy gradient	Speciation gradient	Disturbance gradient	Units
Environmental gradient	0–40	0–40	0–40	0–40	°C
Strength of niche conservatism, σ_E_	1	1	1	1	°C, SD units
Regional carrying capacity, *K_max_*	40,000	4,000–40,000	22,000	22,000	Individuals
Per individual speciation probability	10^-6^	10^-6^	3.2 × 10^-7^ to 3.2 × 10^-6^	10^-6^	Probability
Disturbance magnitude	0	0	0	99–75	% of individuals killed per event

*Values that are listed as a range refer to the values at the temperate and tropical ends of the spatial gradient, respectively, and all gradients were linear*.

For comparison, we also studied a scenario in which the zero-sum constraint was removed. In this case there are no energetic limits and as a consequence there is exponential and indefinite accumulation of individuals and species (Hurlbert and Stegen, 2014). We refer to this as the “pure niche conservatism” scenario, and associated simulations were stopped once total extant richness exceeded 10,000 species (typically a few 100 time steps) for reasons of computational efficiency. Code for running simulations is provided in our online github repository (http://github.com/ahhurlbert/species-energy-simulation).

### SIMULATION METRICS AND ANALYSIS

We examined several simulation metrics that were previously found (Hurlbert and Stegen, 2014) to be helpful in diagnosing processes underlying species richness gradients. These include the correlation between latitude and regional species richness, the correlation between the length of time a clade has been in a region (estimated from its extant members) and regional richness, a measure of phylogenetic tree imbalance or asymmetry (β, Blum and François, 2006), and the slope of the relationship between the scaled mean root distance (MRD) of species in a region and regional richness (Hurlbert and Stegen, 2014).

We also used Bayesian analysis of macroevolutionary mixtures (BAMM, Rabosky, 2014) version 1.0 to analyze the tempo of speciation dynamics across the phylogeny of extant species generated under each scenario. We focused on simulations with an ancestral species in the tropics to be consistent with a tropical region of origin that appears to be common for most large clades (Jablonski et al., 2006; Jansson et al., 2013). For zero-sum simulations, we analyzed the extant phylogeny after 30,000 time steps, after equilibrial richness patterns had been achieved. An advantage of the BAMM approach is that it allows for the estimation of heterogeneous rates across the tree, as might be expected if subclades develop key innovations or colonize previously unoccupied regions. In addition, the method allows for a mixture of diversity-dependent and diversity-independent dynamics across the tree (Rabosky, 2014).

We ran BAMM analyses using phylogeny-specific priors suggested by the setBAMMpriors function in the R package BAMMtools (Rabosky et al., 2014), running 2–10 million generations of reverse jump Markov Chain Monte Carlo sampling depending on the scenario, and discarding the first 20% of generations as a burn in period. Convergence was assessed by comparing 3–5 BAMM runs for each simulated scenario, and re-running BAMM for longer if runs appeared not to have converged. These analyses result in the estimation of marginal probabilities of speciation for each branch in the phylogenetic tree, including an estimate of instantaneous speciation rate at the tips (Rabosky et al., 2014).

## RESULTS

**FIGURE 1:**
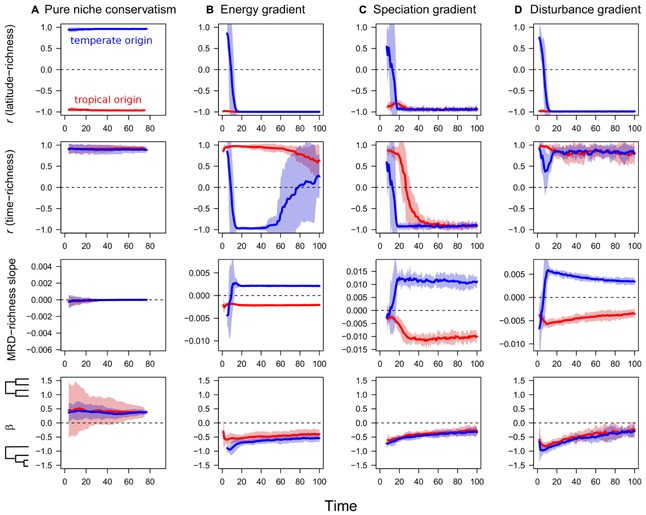
**Temporal trajectories of four metrics of diversity and phylogenetic structure over the course of diversification in four distinct scenarios.** **(A)** Pure niche conservatism, **(B)** energy gradient, **(C)** speciation gradient, and **(D)** disturbance gradient. Metrics include Pearson’s correlation coefficient between latitude and richness, Pearson’s correlation coefficient between the time a clade has occupied a region and richness, the slope of the relationship between mean root distance (MRD) and richness, scaled by maximum possible root distance of the phylogeny, and β, a metric of tree imbalance. Simulations of tropical origin in red, and simulations of temperate origin in blue.

The absence of a zero sum constraint as implemented in the pure niche conservatism scenario left a clear signature in the dynamics of diversification (**Figure 1A**). Clades originating in the tropics developed a strong classical latitudinal gradient (*r* = -1.0), while clades originating in the temperate-most region developed a reverse gradient (*r* = +1.0; **Figure 1A**). As predicted by the niche conservatism hypothesis, independent of region of origin, a strong time-for-speciation effect emerged with the most species in regions that had been occupied the longest (**Figure 1A**). In contrast to all three zero sum scenarios, the MRD-richness slope for the pure niche conservatism scenario was always close to 0 (**Figure 1A**), while β was typically positive, indicating a slightly more balanced phylogeny than expected from random (**Figure 1A**).

The three zero sum scenarios examined—energy gradient, speciation gradient, and disturbance gradient—all shared several features in their simulation output. First, all three scenarios resulted in classical latitudinal gradients regardless of the ancestral region of origin (**Figures 1B–D**). Reverse gradients in richness existed briefly under a temperate ancestral origin, but flipped to traditional gradients within 15–20 thousand time steps. All three scenarios exhibited non-zero MRD-richness slopes, with simulations of a temperate origin yielding positive values and simulations of a tropical origin yielding negative values (**Figures 1B–D**). In addition, all three scenarios resulted in imbalanced phylogenetic trees with negative β, although β appeared to become less negative through time (**Figures 1B–D**).

The time-richness relationship and its dependence on ancestral region of origin was one metric that differed among the three zero sum scenarios. Under the disturbance scenario, a strong, positive time-richness relationship emerged regardless of region of origin (**Figure 1D**). A strong, positive time-richness relationship emerged under the energy gradient scenario, but only for simulations of tropical origin. Under a temperate origin, an initial positive time-richness relationship quickly flipped to be negative as species accumulated in the high energy regions that were colonized last (**Figure 1B**). Later in the simulation as old species in the ancestral region went extinct and were replaced by younger taxa, the strength of the time-richness correlation became weaker. Finally, under a speciation gradient, initially positive time-richness relationships for both regions of origin shifted through time to become negative (**Figure 1C**). This occurred because one result of high speciation rates under a zero sum constraint is high species turnover, resulting eventually in the loss of old basal species and the accumulation of relatively young species in the tropics.

**FIGURE 2:**
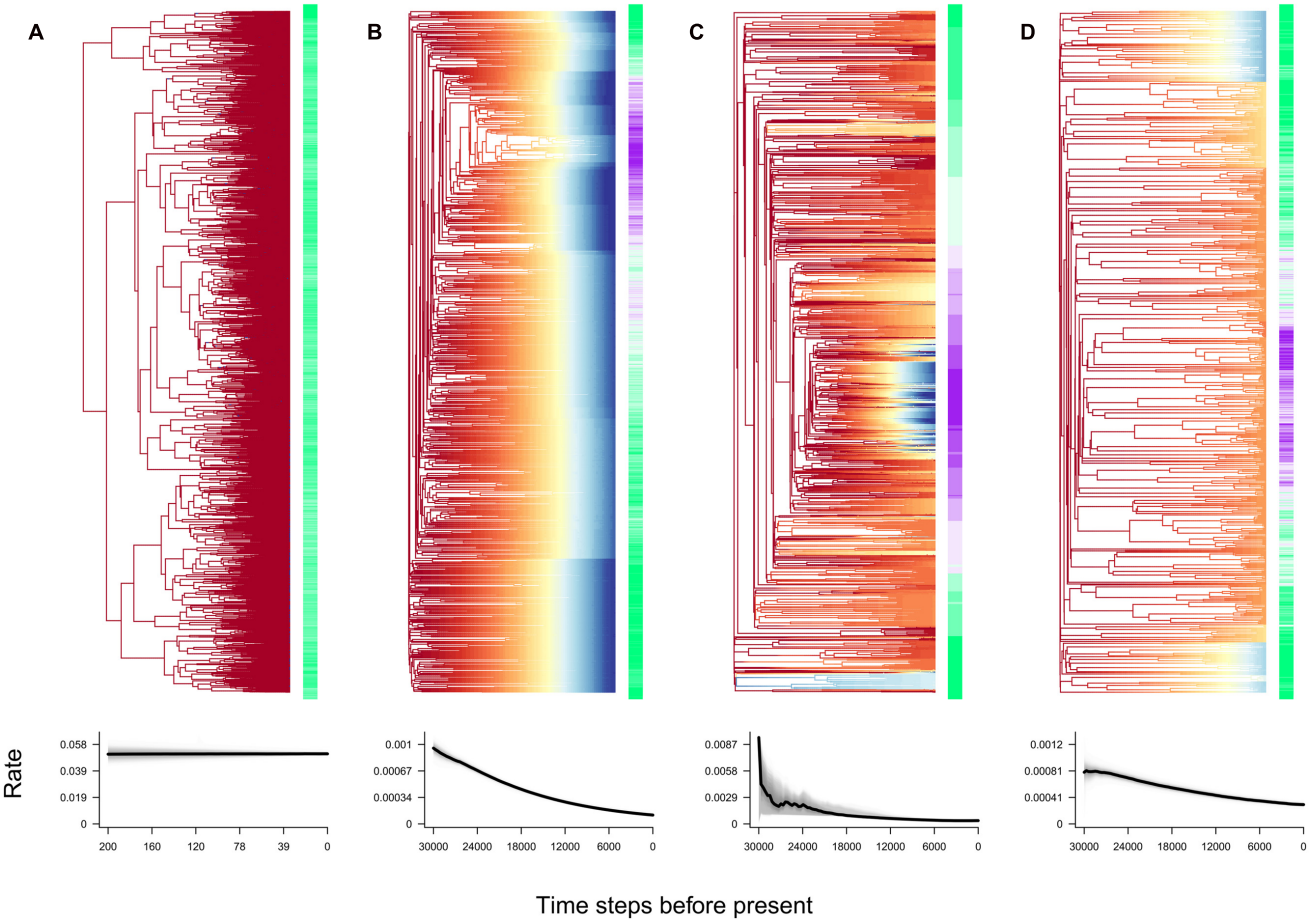
**Output from Bayesian analysis of macroevolutionary mixtures (BAMM) analysis of four distinct diversification scenarios. (A)** Pure niche conservatism, **(B)** energy gradient, **(C)** speciation gradient, and **(D)** disturbance gradient. Top row, extant phylogenies at *t* = 200 time steps **(A)**, or *t* = 30,000 time steps **(B–D)**. Branches are color coded by instantaneous estimates of speciation rate, from low (blue) to high (red). Color labels at the tips reflect the geographic region of maximum abundance for each species, from temperate regions (purple) to tropical regions (green). Bottom row, estimated median speciation rate over the time course of diversification under each scenario.

Bayesian analysis of macroevolutionary mixtures analyses result in very distinct patterns of diversification across the four scenarios (**Figure 2**). Under pure niche conservatism, the best fit model to the phylogeny was one involving a homogeneous process of near-constant per-lineage speciation rates (**Figure 2A**). In contrast, the three zero sum scenarios led to slowdowns in the estimated speciation rate from root to tips of 60–90%, and could be differentiated from each other due to varying degrees of rate heterogeneity within their respective phylogenies (**Figures 2B–D**).

For all three zero sum scenarios, the most basal split led to two lineages which continued to diversify in the tropical region of origin, but only one of which eventually colonized the temperate end of the gradient. Rate shifts identified in BAMM analyses coincided with the colonization of novel, more temperate parts of the gradient. In the speciation gradient scenario, subclades colonizing the temperate-most regions with the lowest per-individual probabilities of speciation predictably resulted in depressed per-lineage speciation rates (**Figure 2C**). In contrast, the disturbance gradient scenario resulted in higher per-lineage speciation rates in temperate regions as disturbance-caused extinctions provided continued opportunities for diversification relative to the more stable tropics (**Figure 2D**).

**FIGURE 3:**
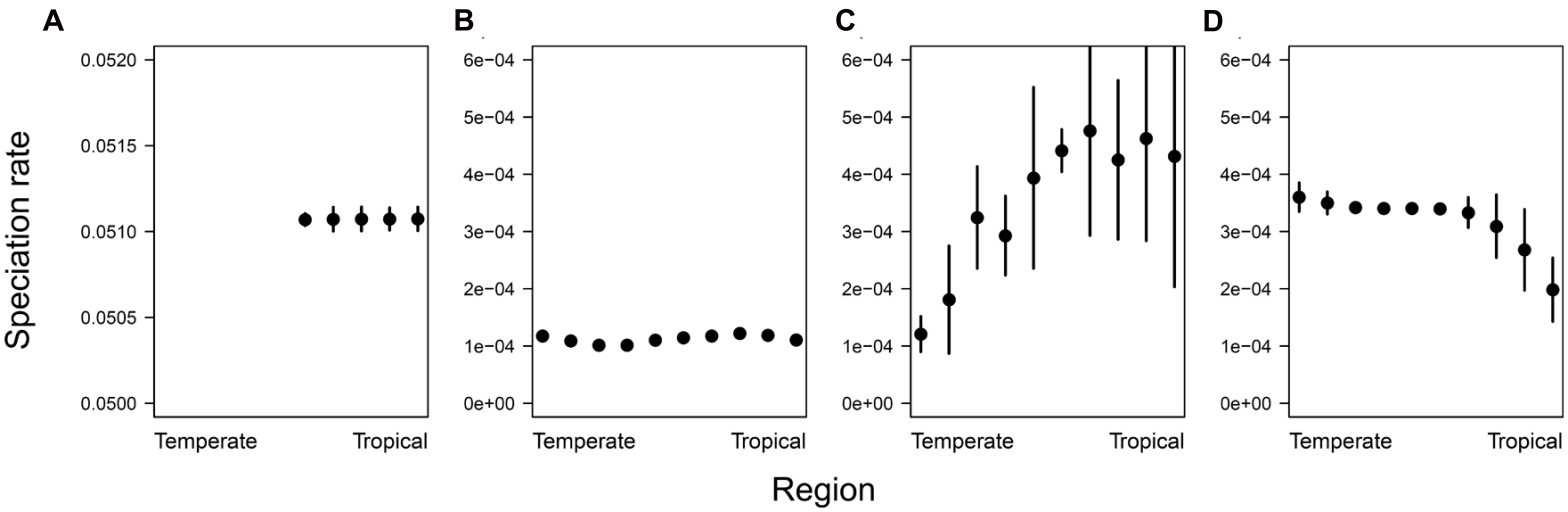
**In each region, the mean of instantaneous tip-level speciation rate estimates was averaged across all species present in that region.** Error bars indicate ±1 SD. **(A)** Pure niche conservatism, **(B)** energy gradient, **(C)** speciation gradient, and **(D)** disturbance gradient. In the pure niche conservatism scenario, the simulation was stopped after 10,000 species had accumulated, at which point species had not yet spread to the temperate-most regions of the gradient.

Averaging tip-specific BAMM estimates of speciation rate across species within regions more directly illustrated these findings. Under the speciation gradient scenario, these rates decreased with latitude, under the disturbance scenario they increased with latitude, and for both the energy gradient and pure niche conservatism scenarios, speciation rate appeared to be independent of latitude (**Figure 3**). This figure also highlights one limitation with our simulations, namely that under the pure niche conservatism scenario, the number of species quickly increased to levels that became computationally intractable to deal with, and hence we were forced to end the simulation prior to colonization of the temperate-most regions. Nevertheless, the trajectory of our metrics over the course of the simulation suggests that basic patterns of diversification and phylogenetic shape would remain unchanged for longer runs that spanned more of the gradient (**Figure 1A**).

## DISCUSSION

Disentangling the relative support for multiple biodiversity hypotheses is challenging because similar patterns may emerge from very different underlying processes. Here, we add to the findings of Hurlbert and Stegen (2014) by identifying several aspects of phylogenetic structure that distinguish a non-zero sum diversification scenario from a set of zero sum scenarios, and further identify speciation rate patterns that distinguish among those zero sum scenarios.

### DIAGNOSTIC VERSUS NON-DIAGNOSTIC BIODIVERSITY PATTERNS

Diversification in the absence of a zero sum constraint—as in the pure niche conservatism scenario—leads to balanced phylogenetic trees with no strong relationship between the MRD in a region and the number of species in that region. In addition, under niche conservatism alone, BAMM analyses show that speciation rate is consistent through time and across clades. This is in contrast to the zero sum scenarios we investigated, which all resulted in imbalanced trees (see also Davies et al., 2011), clear relationships between MRD and richness, decelerating rates of speciation through time, and substantial rate heterogeneity.

Many of the patterns that have been suggested to provide evidence for a non-zero sum, pure niche conservatism explanation of diversity gradients also emerged from the zero sum scenarios. For example, the “time-for-speciation effect” (Stephens and Wiens, 2003; Wiens, 2011), or the relationship between time in a region and number of species in that region, is held as a hallmark for purely historical processes. However, we found a positive time-richness relationship under the disturbance gradient scenario and the energy gradient scenario with a tropical origin. This makes the time-richness relationship a necessary but insufficient test of the tropical conservatism hypothesis—it is unable to rule out alternative hypotheses.

Two additional patterns—beyond time-richness relationships—that have been suggested from verbal arguments to reflect pure niche conservatism did not emerge under our pure niche conservatism scenario. First is the prediction that climate-richness (and hence latitude-richness) relationships should be more variable among subclades under pure niche conservatism, and more similar under ecological constraints (Buckley et al., 2010). In earlier work comparing the same pure niche conservatism and energy gradient scenarios considered here, we showed that in fact the opposite should be expected (Figure 3 in Hurlbert and Stegen, 2014). For small subclades the assumption of a zero sum constraint is more likely to be violated, and hence diversity patterns will not necessarily track aggregate estimates of environmental variation such as net primary productivity (Hurlbert and Stegen, 2014). Second, Hawkins et al. (2006) and Hawkins (2010) have suggested that a higher MRD in temperate regions of low richness—thus, a positive correlation between MRD and richness—was predicted by a pure niche conservatism scenario. Here and in Hurlbert and Stegen (2014), we found the opposite to be true; under pure niche conservatism the MRD-richness slope is expected to be close to 0, while non-zero slopes are expected only if niche conservatism operates alongside a zero sum constraint. Significant correlations between MRD and richness do not, therefore, support the hypothesis that niche conservatism alone is responsible for richness gradients (Algar et al., 2009), and in fact appear to reject that hypothesis.

Our simulations show that the presence of a zero sum constraint results in surprisingly consistent phylogenetic patterns regardless of whether the primary biodiversity driver was limiting resources, speciation rate, or disturbance (**Figure 1**). This provides a broader base of support—expanding results in Hurlbert and Stegen (2014)—for using a combination of the MRD-richness relationship and the value of β to distinguish between zero sum and non-zero sum scenarios within empirical systems. Our simulations further suggest that one can differentiate among zero sum scenarios using BAMM (Rabosky, 2014) to make higher resolution inferences within and across phylogenies. We specifically found that the relationship between mean speciation rate and latitude differs diagnostically among the three zero sum scenarios.

Bayesian analysis of macroevolutionary mixtures analyses revealed that for empirical systems in which there is an overarching zero sum constraint, the observation that speciation rates are highest in temperate regions is sufficient to identify a diversification scenario involving greater magnitudes of disturbance in the temperate zone relative to tropical regions. While by definition disturbance is a non-equilibrial process, the zero sum constraint leads to an equilibrium between speciation and extinction reflecting high taxonomic turnover. Consistent with the disturbance scenario, Weir and Schluter (2007) found speciation and extinction rates of birds and mammals to be higher in northern latitudes relative to rates within the tropics. If it were shown that a zero sum constraint existed for the groups studied by Weir and Schluter (2007)—using β and the MRD-richness slope—and if the latitudinal pattern in speciation rates they observed was also found using BAMM, one could cleanly reject all hypotheses studied here except the tropical stability hypothesis.

The BAMM analyses further revealed that empirical observations consistent with a zero sum constraint and with speciation rates increasing toward the tropics are sufficient to identify a scenario in which a latitudinal richness gradient is driven by increased per-capita speciation probabilities in the tropics. From a theoretical perspective this aligns with the hypothesized kinetic effects of temperature on per-capita evolutionary rates under the metabolic theory of ecology (Allen et al., 2006; Stegen et al., 2009, 2012). Empirically, Rolland et al. (2014) found mammalian speciation rates to be higher in the tropics relative to the temperate zone, and highlighted a variety of processes that may underlie increased speciation rates in the tropics. We suggest that pursuing specific, complementary analyses found here to be diagnostic—MRD-richness, β, and BAMM—would allow evaluation of these underlying processes.

Finally, BAMM analyses showed that empirical evidence for a zero sum constraint but no clear relationship between speciation rate and latitude is consistent with a diversity gradient resulting from a geographic gradient in available energy. Jetz et al. (2012) compiled and analyzed a phylogeny of all 9,993 extant birds and found no relationship between latitude and net diversification rate, although they did not attempt to estimate speciation and extinction rates separately. The lack of a relationship between latitude and speciation rates is the weakest diagnostic in that it may be observed due to lack of power, or to the interaction of conflicting processes. However, an examination of **Figure 2B** indicates that another potentially useful diagnostic is that the variation in estimated speciation rates from root to tips is far greater than the variation among tips. In the other two zero sum scenarios, rate variation from root to tips appears to be of similar magnitude as rate variation among tips. This is a pattern that deserves additional scrutiny in future studies.

### TOWARD A MULTI-HYPOTHESIS, MULTI-PATTERN PARADIGM

Our aim is to emphasize and enable an approach to biodiversity science that has the potential to accelerate progress by using multi-pattern ‘fingerprints’—generated through *a priori* simulation modeling—that can differentiate among alternative hypotheses attempting to explain species richness patterns. We argue that such a shift toward a multi-hypothesis, multi-pattern paradigm is needed as most biodiversity studies evaluate the predictions of a single diversity hypothesis that are often insufficient for ruling out alternative hypotheses. This may be especially important when the interaction between multiple hypotheses is important in driving observed patterns. Unlike the blind men who each focused on the single pattern that was most obvious to them, biodiversity scientists must expand their awareness to consider the breadth and complexity of empirical patterns that only when considered together will reveal the true elephant.

We urge a focus on the comparison of multiple hypotheses using predictions that are diagnostic, rather than predictions that are merely consistent with one particular hypothesis under consideration. The best way to identify diagnostic predictions is to compare secondary biodiversity patterns expected under the modeling of different macroecological and macroevolutionary scenarios (Grimm et al., 2005; Gotelli et al., 2009). We further propose an integrated approach that extends analyses of our simulation model to a larger suite of methods used across empirical studies (e.g., GeoSSE, Goldberg et al., 2011) while simultaneously characterizing empirical systems using the metrics and methods shown here to provide diagnostic signatures. While the comparison of empirical patterns to patterns simulated under different processes has been widely used in macroevolution and phylogenetics (Morlon, 2014), until now no models have included an explicit spatial context (beyond binary tropical versus temperate bins) while simultaneously modeling trait evolution, environmental filtering, and an individual-based energetic constraint (see Appendix 1 in Hurlbert and Stegen, 2014). Our simulation framework thus has the flexibility to model a range of diversification scenarios over spatial and environmental gradients.

Any identification of diagnostic predictions using our approach is provisional. Future work modeling diversification scenarios not examined here could result in duplicate patterns. We view scenarios that invoke the evolution of key innovations that incorporate additional traits related to trophic niche, or that incorporate temporal variation in climate across the different regions as particularly important to consider in future expansions of our model. On the other hand, the identification of patterns as being non-diagnostic is inherently useful and reduces the likelihood that evidence is over-interpreted in favor of one hypothesis over another, regardless of the number of unmodeled scenarios.

In addition, alternative choices in the construction of a simulation model may result in different patterns that can be considered diagnostic. While we have done basic sensitivity analyses to confirm that our conclusions are not dependent on the specific parameter values chosen (see Hurlbert and Stegen, 2014), we welcome the development of alternative simulation models. Testing the robustness of our identified diagnostic patterns to a variety of simulation implementations will provide the strongest support for inferences made from empirical data.

Here we have argued for an approach that generates diagnostic *a priori* predictions across a suite of hypotheses, each associated with a feature of the environment that varies spatially. Our implementation of this approach has focused on generating a set of predictions from each hypothesis and then comparing predictions across hypotheses to identify a diagnostic set of patterns. We recognize, however, that multiple processes contribute to empirical richness gradients. Thus, an important next step is to determine how best to identify the operation and relative importance of multiple processes acting simultaneously to influence richness gradients. As one option for taking this next step, our simulation model could be easily modified such that multiple factors co-vary across the spatial gradient. We encourage such inquiry – our simulation code is publicly available – and further encourage the analysis of alternative simulation models; by integrating multiple models we can triangulate upon patterns that provide the most rigorous hypothesis tests and thereby enable the greatest conceptual advances.

## AUTHOR CONTRIBUTIONS

Allen H. Hurlbert and James C. Stegen designed simulations, Allen H. Hurlbert conducted analyses, and Allen H. Hurlbert and James C. Stegen wrote the paper.

## Conflict of Interest Statement

The authors declare that the research was conducted in the absence of any commercial or financial relationships that could be construed as a potential conflict of interest.
